# The Esculapean

**Published:** 1853-02

**Authors:** 


*The Esculapean.*—Dr. C. D. Griswold has commenced a monthly publication called the *Esculapean*, the object of which is to diffuse information relative to medicine, and the medical profession, among the people. Of course it is designed for popular readers. The following list of headings of the several articles contained in the first No., will convey an idea of the scope of the work: "Sketches from the everyday experience of a Young Doctor;" "How benevolent persons may be led to do evil;" "Water-cure waxes mighty;" "Familiar Letters on the Structure and Functions of the Human System" (with illustrations); "The Chemistry of the Atmosphere;" "January, its joys and sorrows;" "Habit becomes imperative;" "What has become of the Bloomers;" "What every one should know;" "Book notices."

It is published in a folio form with a tripartite division of columns.

As the work costs but a dollar *per annum*, we would advise our readers to subscribe for a year, and if they find it calculated to do good, their inclinations will of course prompt them to use exertions to promote its circulation among the people.

